# College and Hospital Notes

**Published:** 1900-04

**Authors:** 


### COLLEGE AND HOSPITAL NOTES.

The Medical Department of the University of Buffalo will hold its 54th annual commencement at Convention Hall, Friday evening, April 27, 1900, at 8 o'clock. The usual ceremonies with reference to conferring degrees will take place under the supervision of the Chancellor, Hon. James O. Putnam, and the Dean of the Faculty, Dr. Matthew D. Mann. The address to the graduates will be delivered by Daniel B. St. John Roosa, M.D., LL.D., of New York, president of the New York Post-Graduate Medical School and Hospital.

The morning of commencement day will be given over to examinations by the curators of candidates for degrees, of which latter there are fifty or more, and to the class reunions of the Alumni. In the afternoon the annual alumni exercises will take place at Alumni Hall, University Building. These will comprise among other things, papers or addresses by Drs. George Ben Johnston, of Richmond, Va., Lewis S. McMurtry, of Louisville, Ky., and Charles A. L. Reed, of Cincinnati, O. The Alumni meeting is open to the general public, and physicians, undergraduates, ladies and citizens in general who may be interested, will be cordially welcomed. It is desired by the executive committee to make the final meeting of the century a memorable one, and the alumni are requested, by their presence in large numbers, to strengthen the hands of the committee in carrying to successful conclusion their earnest work.

---

THE New Orleans Polyclinic has been reported suspended on account of smallpox in New Orleans. Dr. Isadore Dyer, the secretary, says this statement is absolutely false. The Polyclinic has had a continuous course since November 20, 1899, and will continue until May 12, 1900. The smallpox situation in New Orleans has at no time justified any apprehension on the part of students attending the Polyclinic or on the part of those who might wish to do so.

---

THE burning of the pavilion for consumptives at the Erie County Hospital, on the night of March 21, 190c, was most unfortunate in that it deprives a large number of sufferers of the benefit of that institution. While no lives were lost at the fire it is probable that the exposure incident to escaping from the burning building will shorten the days of some of the patients.

The building was of wood and burned so rapidly that some of the inmates barely escaped injury or worse. The lesson is a dear one, but it means that no building should be constructed for hospital purposes that does not meet all the modern requirements of safety from fire.

---

THE medical staff of the Buffalo Hospital of the Sisters of Charity has been reorganised. At a meeting recently held Dr. Stephen Y. Howell was chosen president; Dr. Lawrence G. Hanley, vice-president; and Dr. C. M. Daniels, secretary. An executive committee was created consisting of Drs. W. H. Slacer, Byron H. Daggett and Eugene A. Smith.

A training school for orderlies and male nurses has been organised at the hospital under the supervision of Dr. B. H. Daggett, who delivered an introductory lecture. There will be two lectures each week by various members of the staff of physicians and surgeons.

